# REFLECTIONS ON VA TRAINING AND EMPLOYMENT FROM INTERVIEWS ON CAREER PATHS IN GEROPSYCHOLOGY

**DOI:** 10.1093/geroni/igz038.038

**Published:** 2019-11-08

**Authors:** Elizabeth M Shumaker, M Lindsey Jacobs, Jessica V Strong, Rebecca S Allen

**Affiliations:** 1 San Francisco VA Health Care System, San Francisco, California, United States; 2 VA Boston Healthcare System, Boston, Massachusetts, United States; 3 University of Alabama, Tuscaloosa, Alabama, United States

## Abstract

The U.S. Department of Veterans Affairs (VA) is one of the largest trainers and employers of psychologists in the world and many clinical geropsychologists have worked for the VA for some portion if not all of their careers. The current qualitative analysis was conducted on phone interviews with 28 psychologists and psychology trainees regarding careers in geropsychology that focused on participants’ reflections on factors that influenced their career paths. This presentation will summarize subthemes from the many participant responses that included statements about the VA. Examples include discussion of the convenience and comfort of transitioning to a VA job after training within this system as well as reflections about the timing of professional development conversations with supervisors and mentors on VA internships and fellowships given the proximity to entering the workforce. Identified benefits and challenges of work in the VA will also be discussed (e.g., varied roles and responsibilities).

